# Seasonality of Kawasaki Disease: A Global Perspective

**DOI:** 10.1371/journal.pone.0074529

**Published:** 2013-09-18

**Authors:** Jane C. Burns, Lauren Herzog, Olivia Fabri, Adriana H. Tremoulet, Xavier Rodó, Ritei Uehara, David Burgner, Emelia Bainto, David Pierce, Mary Tyree, Daniel Cayan, for the Kawasaki Disease Global Climate Consortium

**Affiliations:** 1 Department of Pediatrics, Rady Children’s Hospital San Diego and University of California San Diego, La Jolla, California, United States of America; 2 ICREA and Institut Català de Ciències del Clima (IC3), Barcelona, Catalonia, Spain; 3 Jichi Medical School, Tochigi, Japan; 4 Murdoch Children’s Research Institute, the Royal Children’s Hospital, Parkville, Victoria, Australia; 5 Climate, Atmospheric Science, and Physical Oceanography, Scripps Institution of Oceanography, UCSD and Water Resources Discipline, La Jolla, California, United States of America; 6 US Geological Survey, La Jolla, California, United States of America; University of Florida, United States of America

## Abstract

**Background:**

Understanding global seasonal patterns of Kawasaki disease (KD) may provide insight into the etiology of this vasculitis that is now the most common cause of acquired heart disease in children in developed countries worldwide.

**Methods:**

Data from 1970-2012 from 25 countries distributed over the globe were analyzed for seasonality. The number of KD cases from each location was normalized to minimize the influence of greater numbers from certain locations. The presence of seasonal variation of KD at the individual locations was evaluated using three different tests: time series modeling, spectral analysis, and a Monte Carlo technique.

**Results:**

A defined seasonal structure emerged demonstrating broad coherence in fluctuations in KD cases across the Northern Hemisphere extra-tropical latitudes. In the extra-tropical latitudes of the Northern Hemisphere, KD case numbers were highest in January through March and approximately 40% higher than in the months of lowest case numbers from August through October. Datasets were much sparser in the tropics and the Southern Hemisphere extra-tropics and statistical significance of the seasonality tests was weak, but suggested a maximum in May through June, with approximately 30% higher number of cases than in the least active months of February, March and October. The seasonal pattern in the Northern Hemisphere extra-tropics was consistent across the first and second halves of the sample period.

**Conclusion:**

Using the first global KD time series, analysis of sites located in the Northern Hemisphere extra-tropics revealed statistically significant and consistent seasonal fluctuations in KD case numbers with high numbers in winter and low numbers in late summer and fall. Neither the tropics nor the Southern Hemisphere extra-tropics registered a statistically significant aggregate seasonal cycle. These data suggest a seasonal exposure to a KD agent that operates over large geographic regions and is concentrated during winter months in the Northern Hemisphere extra-tropics.

## Introduction

The search for the causative agent for Kawasaki disease (KD) has now spanned four decades and the environmental trigger for this self-limited pediatric vasculitis remains elusive [1]. Previous epidemiologic investigations suggest that the agent is widely distributed in the environment, that there is no person-to-person transmission, and that genetic susceptibility explains at least some of the variation in disease incidence among different ethnic and racial groups [2-4]. KD was first described by Tomisaku Kawasaki in Japanese children in the early 1960s [5,6]. Since that time KD has been recognized in children of all racial groups from all continents [7]. Japan continues to be the country of highest incidence with an attack rate of 240 per 100,000 children less than 5 years of age [8]. A distinct seasonality has been documented in Japan, Hawaii and San Diego, California, with peaks in late winter and early spring and a nadir in disease activity in the fall [9]. A recent study linked seasonal variations in wind patterns with fluctuations in KD case numbers across the North Pacific from Japan to California. We report here a comprehensive analysis to detect seasonal cycles in the occurrence of KD cases from records that span most of the inhabited parts of the globe. The time series from locations in the Northern Hemisphere extra-tropics revealed a clear seasonal cycle in KD case numbers with most sites having peaks in December through March, with a similar but non-significant trend in the Southern Hemisphere in fall and early winter. This result supports the hypothesis that an environmental trigger operates over a hemispheric scale and leads to seasonal clustering of KD cases.

## Methods

**Figure 1:**
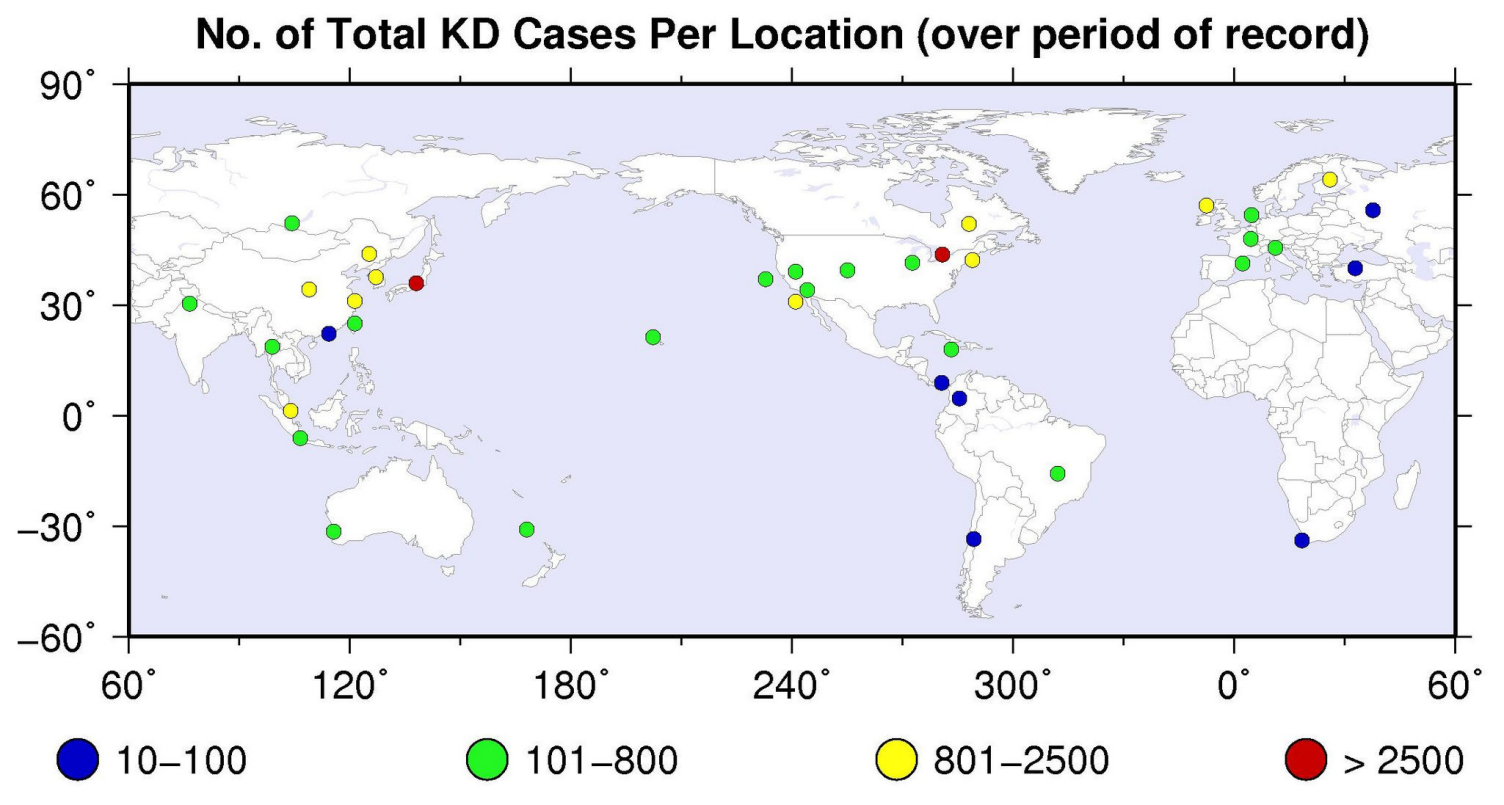
Distribution of KD time series across the globe. Colors indicate number of cases reported in the entire time series from each location.

Collaborating sites were recruited by the following methods: 1) Announcement of the project at the 9^th^ and 10^th^ International KD Symposia held in Taipei, Taiwan and Kyoto, Japan, respectively; 2) E-mail invitation to corresponding authors of English language epidemiologic reports of KD published since 2000 and ascertained through PubMed. Patients included in the time series were those meeting the 2004 American Heart Association (AHA) criteria as follows: 1) ≥ 3 days of fever and 4/5 classical clinical criteria or 2) ≥ 5 days of fever, fewer than 4 criteria, but dilated or aneurysmal coronary arteries as defined by AHA criteria [10]. Time series from locations that contained less than 30 cases (2 countries: Panama and Columbia) were eliminated from the analysis because the number of samples was too small to yield a stable estimate of any seasonal variation that might occur. The remaining 296,203 KD cases were assembled from 39 locations in 25 countries: 27 locations in the extra-tropical Northern Hemisphere, 8 in the tropics, and 4 in the extra-tropical Southern Hemisphere (Table **1**, Figure **1**). The individual time series represented different blocks of years that collectively covered the period 1970-2012. The minimum number of cases was 30 from Ankara, Turkey, and the maximum number of cases was 271,475 from Japan.

**Table 1:** International Kawasaki Disease Climate Consortium.

**Country**	**Location**	**Period of Record**	**Number of Cases**	**Dataset Contributors**	**Affiliations**
***NorthernHemisphereextra-tropics***					
Canada	Montreal	3/1980 - 3/2009	1,382	N. Dahdah, M. Gibbon, R. Scuccimarri	Sainte-Justine University Hospital Center, Montreal Children’s Hospital/McGill University Health Centre
	Ontario	12/1994 - 12/2009	2,884	B. McCrindle, C. Manlhiot	The Hospital for Sick Children, University of Toronto
China	Jilin	12/1999 - 12/2010	1,016	C-J. Jin, L-H. Jin, Z-Y Jin, J-H Piao, Y Zhou	Department of Pediatrics, First Affiliated Hospital, Jilin University
	Shaanxi	8/2007 - 4/2011	1,047	F. Jiao	Dept. of Pediatrics, The Shaanxi Provincial People’s Hospital of Xi
	Shanghai	1/1998 - 3/2011	1,748	G-Y. Huang	Pediatric Heart Center, Children’s Hospital of Fudan University
Finland		5/1982 - 12/2010	948	E. Salo	Helsinki University Central Hospital
France	Lyon	6/1979 - 4/2007	211	R. Cimaz, S Di-Filippo, J-C. Lega	Department of Pediatric Cardiology, Louis Pradel Hospital
India	Chandigarh	5/1994 - 1/2011	259	R. Aulakh, S. Singh, D. Suri	Pediatric Allergy Immunology Unit, Advanced Pediatrics Centre, Post Graduate Institute of Medical Education and Research, Chandigarh
Israel		1/1996-12/2009	764	M. Bar-Meir	Pediatrics and Infectious diseases, Shaare-Zedek Medical Center, Jerusalem
Italy	Florence	7/1997 - 6/2009	162	R. Cimaz	Department of Pediatric Rheumatology, Anna Meyer Children’s Hospital
Japan		1/1970 - 10/2010	271,475	Y. Nakamura, R. Uehara	Department of Public Health Jichi Medical University
Korea	Daejeon	1/1987 - 12/2008	772	K-Y. Lee	Department of Pediatrics, The Catholic University of Korea, Daejeon St. Mary’s Hospital
	Uijeoungbu	1/1993 - 12/2008	723	J-W Han	Department of Pediatrics, The Catholic University of Korea, Uijeongbu St. Mary’s Hospital
	Seoul	12/1993 - 4/2009	473	M-K. Han, Y-M. Hong*, G-Y. Jang**, D-S. Kim***, H-D. Lee****, J-K. Lee and I-S. Park*****, M-S. Song******, S-W. Yun*******	Department of Pediatrics, University of Ulsan, Gangneung Asan Hospital,*Department of Pediatrics, Ewha Womans University Hospital,**Department of Pediatrics, Korea University Hospital,***Department of Pediatrics, Yonsei University College of Medicine, Severance Children’s Hospital,****Department of Pediatrics, Pusan National University Hospital,*****University of Ulsan, Asan Medical Center,******Department of Pediatrics, Inje Univeristy, Paik Hospital,*******Department of Pediatrics, Chung-Ang Univeristy Hospital
Netherlands	Amsterdam	1/1987 - 12/2010	501	W.B. Breunis, T.W. Kuijpers, C.E. Tacke	Division of Pediatric Hematology, Immunology and Infectious diseases, Emma Children’s Hospital, Academic Medical Center
Russia	Moscow	1/2008 - 12/2010	64	G. Lyskina, O. Shirniskaya, A. Torbyak	Sechenov First Moscow State Medical University
	Irkutsk	1/2003 - 5/2011	103	L. Bregel, T. Soldatova, V. Subbotin,	Irkutsk State Academia of Continuing Medical Education, Kawasaki Disease Center, Irkutsk Regional Children"s Hospital, Irkutsk Regional Hospital
Spain	Barcelona	6/1984 - 1/2009	158	J. Anton, F. Prada*, S. Ricart, R. Bou	Pediatric Rheumatology Unit and * Cardiology Department, Hospital Sant Joan de Déu and Universitat de Barcelona.
Turkey	Ankara	3/1994 - 2/2011	30	S. Atalay*, E. Çiftçi, E. İnce, A. Karbuz, H. Özdemir	Department of Pediatric Infectious Diseases and * Department of Pediatric Cardiology, Ankara University Medical School
UK	UK	8/1982 - 3/2005	2,009	A. Harnden, M. Levin*, R. Mayon-White, R. Tulloh**, C. Michie***, V. Wright*	Department of Primary Health Care Sciences, University of Oxford, Oxford *Pediatrics Faculty of Medicine, Imperial College, London,**Bristol Royal Hospital for Children, Bristol ***Ealing Hospital NHS Trust, London
United States of America	Boston, Massachusetts	4/1976 - 12/2010	1,407	A.L. Baker, J.W. Newburger	Children’s Hospital Boston
	Chicago, Illinois	1/2004 - 12/2010	420	N. Innocentini, S. T. Shulman	Division of Infectious Diseases, Ann and Robert H. Lurie Children’s Hospital of Chicago
	Denver, Colorado	11/1997 - 12/2010	459	M. Anderson, S. Dominguez, M. Glode	Pediatric Infectious Disease Children’s Hospital, Colorado
	Orange, California	12/1993 - 11/2010	731	A. Arrieta	Pediatric Infectious Diseases, Children’s Hospital of Orange, County
	Los Angeles, California	1/2000 - 7/2009	345	C. Dozal, W. Mason	Children’s Hospital of Los Angeles
	Riverside, California	1/1991 - 1/2011	491	J. Beck	Department of Pediatrics, Loma Linda University Medical Center
	San Diego, California	10/1978 - 1/2012	1,274	J. C. Burns, A. Tremoulet, S. Fernandez	Department of Pediatrics, UCSD School of Medicine
***Tropics(between23°Nand23°S***)					
Brazil	Brasilia	2/2002 - 2/2011	154	C.M. Magalhaes, R. Pratesi	Dept. of Pediatrics, Brasilia University School of Medicine
China	Hong Kong	6/2006 - 4/2011	52	Y-F. Cheung	Department of Pediatrics and Adolescent Medicine, Queen Mary Hospital
Colombia	Bogota	2/2006 - 12/2008	13	M. Reyes	Departamento de Microbiología, Pontificia Universidad Javeriana
Indonesia	Jakarta	2/2001 - 7/2009	210	N. Advani	University of Indonesia, Jakarta Pusat
Jamaica		12/1986 - 10/2005	102	O. Olugbuyi, R. Pierre	Department of Child and Adolescent Health, University of the West Indies
Panama		2/2011 - 6/2011	10	E. Castaño, D. Estripeaut and X. Sáez-Llorens	University of Panama School of Medicine, Hospital del Niño
Singapore		1/1998 - 12/2008	1,306	C. K. Chen, T. L. J. Choo, T. H. Tan, K. Y. Wong	Cardiology Service, Department of Pediatric SubspecialtiesKK Women’s & Children’s Hospital
Taiwan	Taipei+Kaohsiung	1/1999 - 12/2010	652	H-C. Kuo, M-T. Lin*, M-H. Wu*	Department of Pediatrics, Kaohsiung Chang Gung Memorial Hospital,*Department of Pediatrics, National Taiwan University Hospital
Thailand	Chiangmai	1/2000-12/2010	237	R. Sittiwangkul	Division of Cardiology, Department of Pediatrics, Chiang Mai University
United States of America	Honolulu, Hawaii	2/1996 - 3/2011	652	M. Melish	Department of Pediatrics, Kapiolani Medical Center
***SouthernHemisphereextra-tropics***					
Australia	Perth	3/1977 - 7/2009	418	D. Burgner, M. Odam	Murdoch Childrens Research Institute and Department of Pediatrics, University of Melbourne
Chile		5/2004 - 12/2009	44	A. Salgado, G. Soza	Dr. Hernan Henriquez Aravena Hospital
New Zealand		7/1995 - 6/2006	441	J. Doran, P. Heaton*, N. Wilson**	Taranaki Base Hospital,*Paediatric Department, Yeovil District Hospital,**Department of Pediatric Cardiology, Starship Hospital
South Africa	Cape Town +Johannesburg	5/2004 - 11/2010	52	B. Eley, D. Moore*	Pediatric Infectious Diseases Unit, Red Cross War Memorial Children’s Hospital and Department of Pediatrics and Child Health, University of Cape Town,*Respiratory and Meningeal Pathogens Research Unit, University of the Witwatersrand

For sites with only the country name listed, the KD cases were collected from the entire country over the time period specified.

For each of the individual time series, the mean number of KD cases for each of the 12 months of the year was obtained by averaging all the cases in that month and dividing by the number of times that month appeared in the time series. First, the time series were plotted and evaluated visually to detect sampling artifacts and other issues. The presence of seasonal variation in the KD records was tested using three different techniques: comparing the fit of seasonal versus non-seasonal time series models, spectral analysis, and a Monte Carlo sampling exercise.

Many of the time series a showed large number of zero reported cases near the beginning of the record. In the time series and spectral analyses, years at the start of the series with fewer than 24 observations were discarded to reduce the effect of these low-sample years on the analysis. This would have eliminated 8 of the stations in their entirety (noted in Table 2), so those stations were allowed to have as few as 12 observations/year. Many of the time series also showed trends with associated heteroscedasticity; to reduce this, the best-fit second order polynomial to the time series was calculated and the data divided by the fitted value. This served both to remove the trend and reduce the disparity of variance over time. One location, Japan, had a very large number of reported cases in three isolated years between 1979 and 1987. The Japanese time series including only years 1990 and later was analyzed in addition to the entire time series, and no differences from the values reported in Table 2 were found. A set of seasonal and non-seasonal autoregressive/moving average (ARMA) models of the autocorrelation structure of the KD series were fit to the KD data [11]. . The best-fit seasonal model of order up to two was compared to the best-fit non-seasonal model of order up to two. Based on the Akaike Information Criterion (AIC) results from the seasonal vs. non-seasonal best-fit models, locations were separated into the following categories: 1) strongly seasonal; the best seasonal model is more than 5 times more likely to be correct than the best non-seasonal model; 2) seasonal: the best seasonal model is between 2 and 5 times as likely to be correct; 3) indeterminate: the relatively likelihood of the best seasonal and non-seasonal models is within a factor of 2; 4) non-seasonal: the best non-seasonal model is between 2 and 5 times as likely to be correct compared to the best seasonal model; 5) strongly non-seasonal if the best non-seasonal model is 5 or more times as likely to be correct as the best seasonal model.

**Table 2:** Seasonality tests of KD monthly time series at each location using ARMA time series model fitting, spectral analysis, and a Monte Carlo simulation to gauge the significance of the highest difference in the average monthly KD incidence.

**Location**	**Seasonality of ARMA time series model**	**Confidence level of annual or biannual Spectral Peak**	**Significance of seasonal differences from Monte Carlo testing**
**Northern Hemisphere extra-tropics**			
Los Angeles, CA, USA	**S**	−	**0.0001**
Orange, CA, USA	**S**	99	**0.0001**
San Diego, CA, USA	**SS**	99	**0.0001**
Riverside, CA, USA	I	−	0.6949
Denver, CO, USA	I	90	**0.0008**
Chicago, IL, USA	N	95	**0.0149**
Toronto, Canada	**SS**	99	**0.0001**
Montreal, Canada	**SS**	99	**0.0001**
Boston, MA, USA	**SS**	99	**0.0001**
UK	**SS**	99	**0.0001**
Amsterdam, Netherlands	N	−	0.4856
Lyon, France^+^	**SS**	90	0.3756
Florence, Italy	N	−	0.0606
Barcelona, Spain^+^	I	−	**0.0957**
Finland	**S**	90	**0.0001**
Moscow, Russia^+^	N	−	0.4141
Ankara, Turkey	−	−	0.7348
Israel	**SS**	95	**0.0006**
Chandigarh, India	I	95	**0.0001**
Irkutsk, Siberia	I	−	0.1884
Shaanxi, China	**S**	95	**0.0001**
Shanghai, China	**SS**	99	**0.0001**
Jilin, China	**SS**	90	**0.0001**
Seoul, Korea	I	90	**0.0004**
Daejeon, Korea	I	−	**0.0079**
Uijeongbu, Korea	**SS**	95	**0.0421**
Japan	**SS**	99	**0.0001**
**Tropics**			
Hawaii, USA	N	−	0.3785
Jamaica^+^	I	−	0.2688
Brasilia, Brazil^+^	**SS**	99	**0.0019**
Chiang Mai, Thailand	N	−	0.3371
Singapore	I	−	0.7492
Jakarta, Indonesia	N	−	**0.0043**
Hong Kong, China^+^	N	−	0.9204
Taipei and Kaohsiung, Taiwan	I	−	**0.0903**
**Southern Hemisphere extra-tropics**			
Temuco, Chile^+^	N	−	0.6891
Cape Town and Johannesburg, S. Africa^+^	I	−	0.3012
Perth, Australia	I	−	0.8096
New Zealand	**S**	−	**0.0354**

ARMA model results are reported for locations whose model fits, according to the Akaike Information Criterion, registered as strongly seasonal (SS), seasonal (S), indeterminate (I), or non-seasonal (N). The statistical significance of annual or biannual (6 months period) spectral peaks was reported if they exceeded the 90^th^, 95^th^ or 99^th^ percent significance level. The statistical significance of the largest monthly mean difference of any two months from the observed series at each location using a ranked set of Monte Carlo simulated monthly mean differences (see text) is reported in bold numerals if the p-value was less than 0.10. ^+^ years with as few as 12 cases per year were included at the start of the time series in the ARMA and spectral analysis; for the other stations, only years with 24 or more cases were included.

The individual KD time series were also tested using spectral analysis implemented using the Fourier transformation of the auto-covariance of the time series [12]. The spectral analysis was performed on the original monthly data as well as the data pre-processed as described above to reduce the zero count and heteroscedasticity. Results from these two forms of the monthly series were similar, but to minimize any influence from the trends, the results from the pre-processed series are reported here. A KD time series was deemed strongly seasonal if it had a spectral peak at the 99% significance level; seasonal if it had a peak between the 90% and 99% significance level; and non-seasonal otherwise. A significant spectral peak at either the annual or biannual frequency was considered to indicate seasonal KD structure.

Each individual KD series was also evaluated for seasonality by comparing the observed series against the distribution of a seasonal measure estimated from 10,000 simulated series generated in a Monte Carlo sampling exercise. The possibility that the differences between means of the 12 months were merely the result of random sampling was evaluated using a Monte Carlo test in which each case in a given record was assigned a month according to a random process and this exercise repeated 10,000 times. For each of these simulated time series, we calculated the mean for each month and subtracted the lowest mean from the highest mean as an indication of the seasonal variation. From the 10,000 simulated series, the set of resulting monthly differences was ranked from lowest to highest. The observed maximum monthly mean difference was then assigned a p-value according to its rank within the simulated ordered set. In order to test the consistency of the seasonal patterns over time, the observed KD time series were tested as a whole and as two subsets by dividing the time series into two halves that were independently tested and compared.

To test whether seasonal variation in the cases occurred systematically across a large spatial domain, two additional analyses were conducted: Hewitt’s statistical measure of seasonality [13] and an empirical test of significance of the observed area-aggregate mean monthly variation against a set of simulated monthly means obtained from a Monte Carlo sampling exercise. If a seasonal structure occurred randomly across locations, the resultant aggregate monthly means would be unlikely to contain statistically significant peaks or troughs. Thus, we stratified our analysis of three geographic regions by aggregating time series over the following broad sectors: the extra-tropics of the Northern Hemisphere, the tropics, and the extra-tropics of the Southern Hemisphere. Extra-tropics were defined as the regions poleward from 23° latitude in the Northern and Southern Hemispheres. To ensure that time series from each location had the same weight, the monthly means for each individual time series were normalized by dividing by the overall monthly mean (the total number of cases divided by the total number of months in entire time series). The normalized monthly means were then averaged over each of the three broad geographic sectors including 27 records from the Northern Hemisphere extra-tropics, 8 records from the tropics, and 4 records from the Southern Hemisphere extra-tropics.

Hewitt’s statistic for seasonality in monthly data is a non-parametric test wherein one calculates the maximum rank sum, amongst all possible rank sums of the ranks of consecutive 6 month periods [13]. The probability of occurrence of various values of the maximum rank sum for sequences of 6 months has been tabulated.

Under the Monte Carlo random sampling exercise, the normalized monthly means and their relative sequence were preserved, but the 12-month block was randomly shifted. The simulation was conducted independently for each of the normalized time series in the sector. The resulting monthly means from all the locations in the geographic sector were then averaged, and the largest difference between any two months was identified. This same exercise was performed 10,000 times and the resulting set of 10,000 largest monthly differences was ranked from low to high. The largest difference between any pair of the 12 values of the observed aggregated monthly means was placed into this ranked 10,000 member synthesized set of monthly differences in order to establish its statistical significance (p-value). Time series were tested as a whole and as two subsets by dividing the time series into two equal halves that were independently tested.

## Results

The largest number of records and the highest number of KD case reports was available in the extra-tropical Northern Hemisphere (north of 23° N). A significant seasonal structure was present in many of the records in this zone.

From the ARMA model fitting analysis, of the 27 Northern Hemisphere time series, 11 registered as strongly seasonal and 4 registered as seasonal (Table **2**). No stations fell into the strongly non-seasonal category.

**Figure 2:**
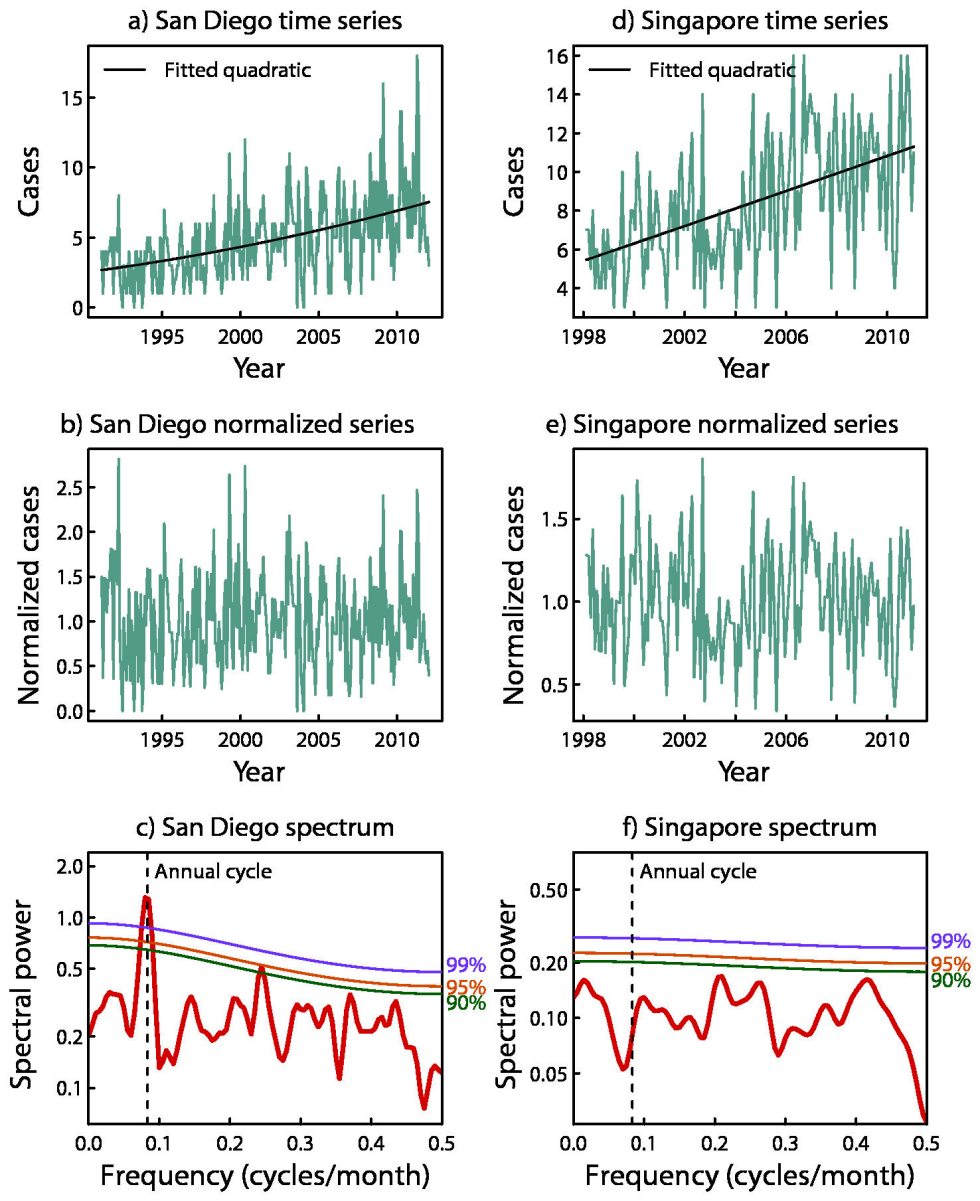
Original time series (top), normalized time series (middle), and spectral analysis (bottom) for San Diego and Singapore. 90^th^, 95^th^ and 99^th^ percentile significance levels shown by green, red and purple lines in bottom frame.

From the spectral analysis, 8 locations exhibited an annual or semi-annual peak whose amplitude exceeded the 99% significance level; 7 of those were found to be strongly seasonal by the ARMA analysis, and the other was found to be seasonal. Five locations exhibited a spectral peak that was less than the 99% level but exceeded the 95% significance level, and five registered a peak that was less than the 95% level but exceeded the 90% significance level (Table **2**). As an example, the original time series, normalized time series, and spectral analysis for two locations, San Diego (which registered a highly significant annual peak), and Singapore (which did not register a significant annual or semi-annual peak) are shown in Figure 2.

Under the individual site Monte Carlo testing, 20 of the 27 records within the Northern Hemisphere extra-tropics registered seasonal change magnitudes with p-values ≤ 0.05 (Table **2**). In addition, this seasonal structure remained significant for all sites when the time series were divided into two halves and analyzed separately using a similar Monte Carlo exercise. Importantly, the locations registering as significant in the other two tests (ARMA model fitting and spectral analysis) were consistent with those obtained from the Monte Carlo exercise. Also, for the two sites with highest numbers of KD cases, KD incidence was estimated using census data to identify the at-risk pediatric population. Seasonality test results using each the three analysis methods were essentially identical to those obtained using the original monthly case occurrence data. Thus we conclude that the KD case occurrence data is an adequate form of the KD sample data to investigate for seasonality. 

**Figure 3:**
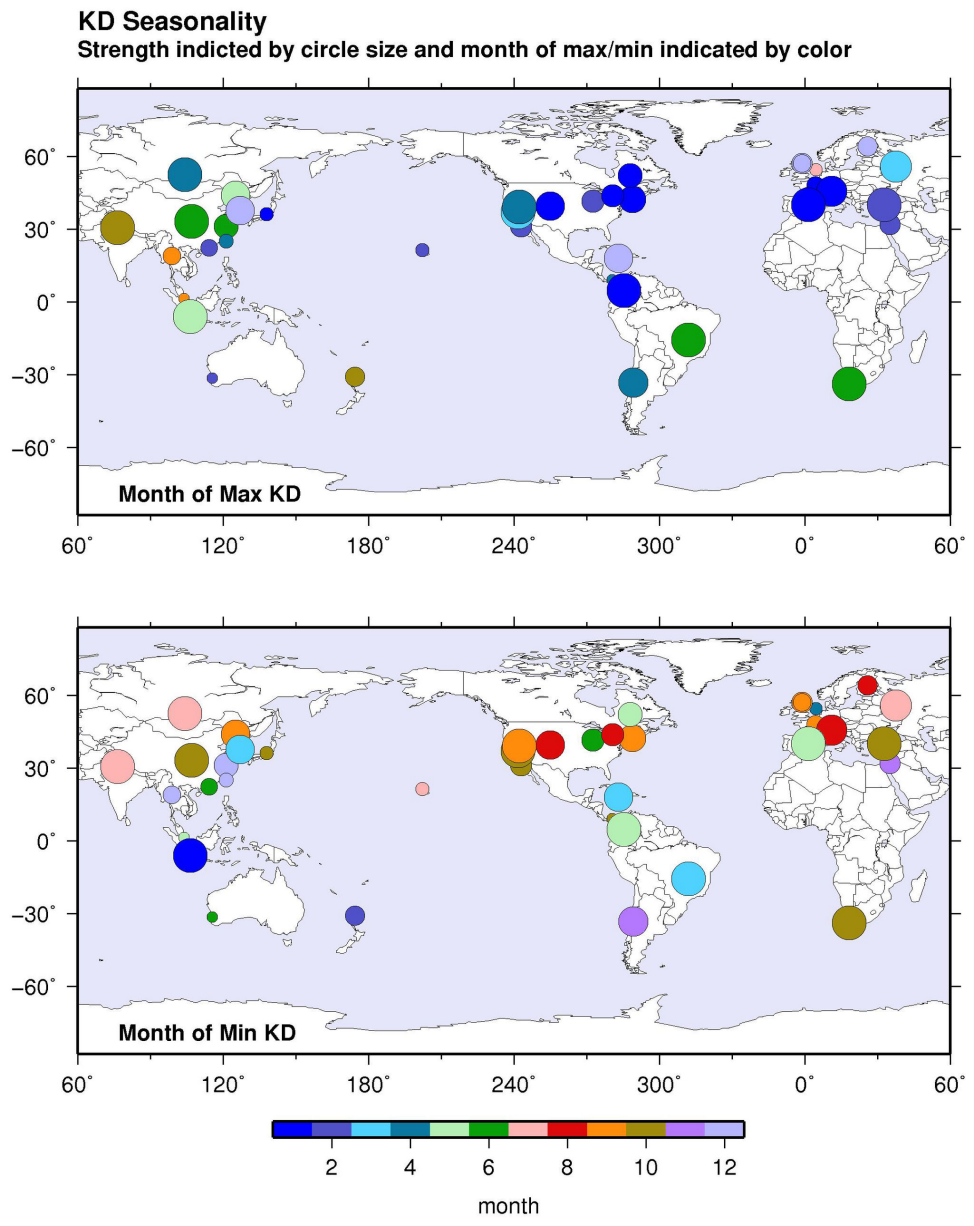
Months of maximum and minimum KD case numbers for each time series. The normalized amplitude of the seasonal difference (maximum case numbers minus minimum case numbers) is indicated by the size of the dot. Month of maximum case numbers (upper map) and month of minimum case numbers (lower map) is indicated by the color of the dot.

**Figure 4:**
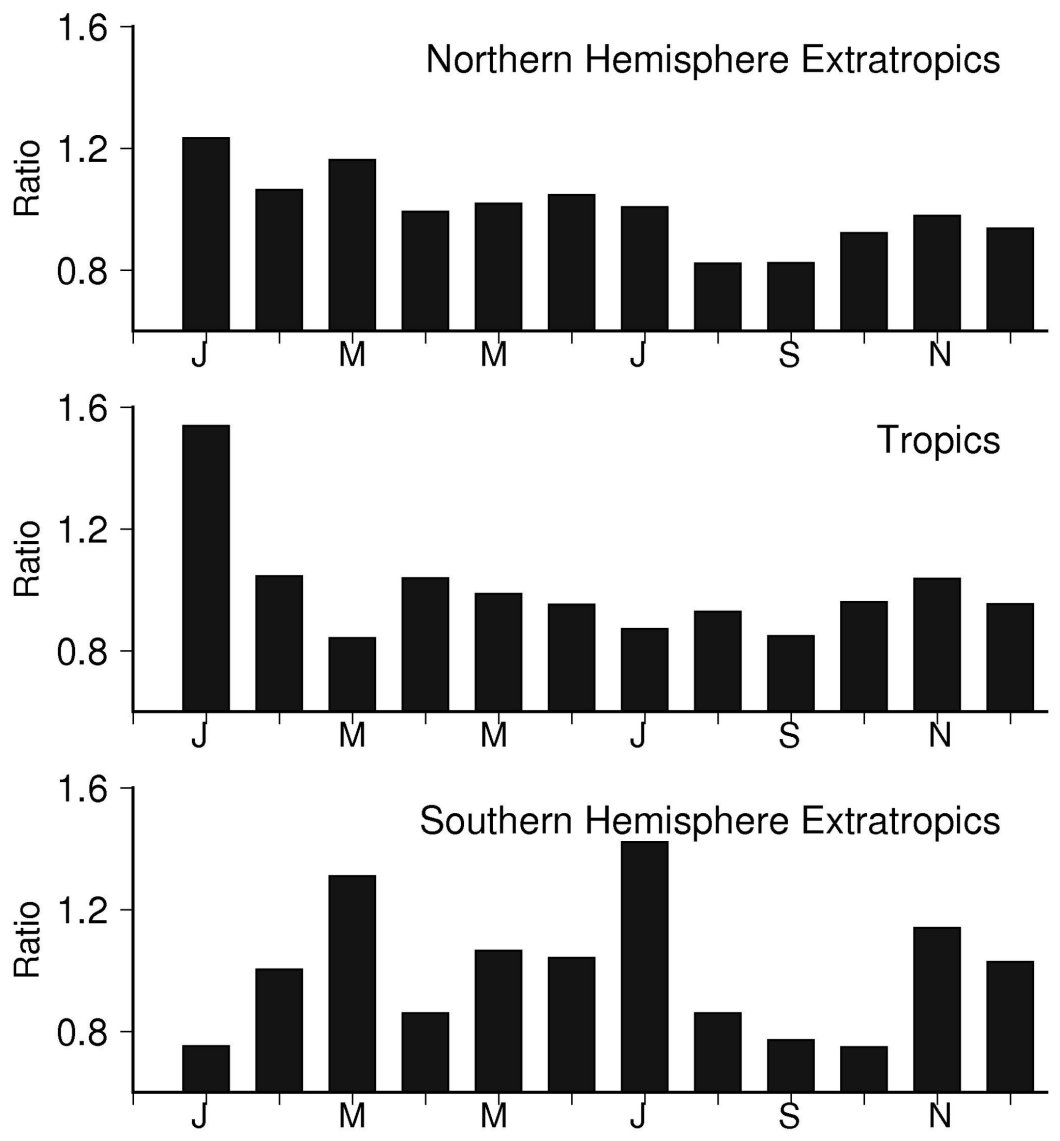
Seasonality of KD by region. Average of the ratio of normalized monthly mean KD cases to overall number of cases per month over a) Northern Hemisphere extra-tropics, b) Tropics, c) Southern Hemisphere extra-tropics.

Looking further, the timing of seasonal peaks and troughs that occurred in locations across this zone was not randomly distributed (Figure **2**). This suggests that a process operates over a hemispheric scale, which results in this seasonal coherence in fluctuations of KD case numbers. As shown by mapping of the seasonal peaks and troughs from the Northern Hemisphere extra-tropical aggregated monthly mean, KD occurrence was highest in January through March and approximately 40% higher than in the months of lowest occurrence from August through October (Figures **3** and **4**). The seasonality of the aggregated Northern Hemisphere KD occurrence proved to be highly significant, as gauged by Hewitt’s statistic and according to the distribution obtained by the Monte Carlo sampling exercise (Table **3**). Furthermore, when the Northern Hemisphere records were divided into the first and second halves of the available time period, the aggregated monthly means from both the first and second halves formed similar seasonal variations, and registered as highly significant under Hewitt’s statistic and from the Monte Carlo distributions.

**Table 3:** Test of seasonality in KD variation aggregated over Northern Hemisphere extra-tropics, Tropics, and Southern Hemisphere extra-tropics, as determined by Hewitt’s statistic and by gauging the observed monthly variation using a distribution created using a Monte Carlo exercise.

**Sector**	**# KD cases**	**Hewitt’s Statistic**		**Monte Carlo**
		**6-month maximal rank sum**	**p-value**	**p-value**
No Hemisphere extra-tropics	291,856	56	**0.025**	**0.002**
Tropics	3,365	49	0.48	0.67
So Hemisphere extra-tropics	955	50	0.37	0.58

The statistical significance of the largest monthly mean difference of any two months from the observed sector-aggregate was assessed from a ranked set of Monte Carlo simulated monthly mean differences.

In the Tropics (23° N to 23° S) and in the Southern Hemisphere extra-tropics (south of 23° S), there were fewer time series and fewer numbers of cases reported. Under each of the three analysis procedures, the KD time series in these zones did not, for the most part, contain distinguishable seasonal variations. Only one of the records registered as strongly seasonal, and one as seasonal, using the ARMA time series analysis. The strongly seasonal location, Brasilia, Brazil, also showed the only significant spectral peak (at the 99% level) of any tropical or Southern Hemisphere location. From the Monte Carlo exercise, 2 of the 8 records in the tropics (including Brasilia) and 1 of the 4 records in the Southern Hemisphere registered seasonal change magnitudes with p-values ≤0.05 (Table **2**). Datasets were much sparser in the Southern Hemisphere compared to the Northern Hemisphere, but suggested a maximum in May through June, with an occurrence approximately 30% higher than in the months of lowest occurrence in February, March and October (Figure **4**). However, neither the Tropics nor the Southern Hemisphere extra-tropics yielded an aggregate seasonal variation with a significant p-value (Table **3**).

## Discussion

In a comprehensive analysis of KD cases from across the globe, many time series from the Northern Hemisphere extra-tropics exhibited statistically significant, seasonal variations in KD activity. There was coherence in the seasonal patterns amongst the Northern Hemisphere records. When their normalized long-term the monthly mean occurrences were averaged together, a statistically significant seasonal cycle was apparent, with peak KD occurrence from January through March and a nadir in KD occurrences during the months of August and September. For the Tropics and Southern Hemisphere extra-tropics, a statistically significant seasonal structure was not evident with these techniques, but detecting a seasonal signal was hampered by sparse sampling and fewer numbers of cases in these zones.

Seasonality test results using the three analysis methods operating on estimated KD incidence rates in Japan and in San Diego were essentially identical to those obtained using the original monthly case occurrence data. Thus we concluded that the KD case occurrence data is an adequate form of the KD sample data to investigate for seasonality.

A seasonal occurrence of KD has long been suspected but this is the first analysis in which a dataset with global coverage has been available. Yanagawa and colleagues were the first to note a seasonal peak in Japan beginning in December and tapering off in March [14]. Subsequently, a more formal analysis of seasonality in Japan and San Diego, California, documented a winter/spring peak followed by a lesser summer peak and a nadir in the fall [9]. Detailed analysis of cases in San Diego suggested both temporal and spatial clustering [15]. Numerous regional case series have echoed this same observation [14,16-19]. The collective dataset assembled here, which includes Japan and San Diego but also records from many other locations, indicates that KD does have a distinct seasonality that is shared by regions distributed across extra-tropical latitudes of the Northern Hemisphere. This observation suggests an environmental trigger for KD that operates on a very large scale.

While environmental exposure appears to play an important role in determining the seasonality of KD, host genetics also influences KD susceptibility. Recent analyses of Asian and European descent populations have uncovered important genetic variation in biologic pathways that shape host susceptibility to KD [20-22]. However, the present data collected from all the inhabited continents of the globe suggest a complex interplay of host genetics and environmental exposure. It has long been recognized that Japan has the highest incidence of KD and that children of Japanese descent residing outside of Japan retain their high susceptibility to the disease [23]. Recently, unique genetic determinants in the human leukocyte antigen (HLA) locus and in the CD40 gene have been shown to influence susceptibility in Asian populations but not in children of European descent [20]. Other shared genetic determinants have also been identified, which helps to account for the fact that KD has been diagnosed in children of all races and ethnicities across the globe [4].

One mechanism that may explain the hemispheric seasonal structure contained in the global KD records is the recent observation that large scale tropospheric wind patterns are associated with fluctuations in KD cases [24]. In an analysis of the three major Japanese KD epidemics that occurred in 1979, 1982, and 1986, a close relationship with directionality of tropospheric winds was observed. When winds blew from the northwest across Japan in a southeasterly direction, the number of KD cases increased. At the conclusion of the epidemics, the wind reversed direction and blew across Japan from the Pacific Ocean in a northwesterly direction. This pattern was repeated on an interannual cycle and anomalously high or low KD case numbers were associated with corresponding high and low intensities of these tropospheric winds. The passage of these large-scale wind patterns across the Pacific Ocean was similarly associated with an increase in KD cases in San Diego. During winter, there are stronger seasonal winds across the Northern Hemisphere and this might lead to greater transport of the putative KD agent that could explain, in part, the observed coherence in KD seasonality.

We recognize some important strengths and limitations of the current analysis. This is the first spatially comprehensive analysis of KD time series, and the results suggest that it is useful to view KD over a global domain to understand disease mechanisms and to survey trends. Clearly, there is a need for more rigorous reporting of KD cases and the creation of comprehensive time series from a greater number of sites to help refine this type of global analysis. The sparse time series with very low numbers from the Tropics and Southern Hemisphere precluded a more robust statistical analysis of seasonality in these locations. Another limitation is that cases were contributed by individual investigators and under-reporting of cases within a given region is likely. Without a gold standard diagnostic test, there was surely misclassification of cases as well as missed cases in all the time series. That said, the remarkable coherence of KD occurrences in the Northern Hemisphere extra-tropics suggests that many of these time series were sufficiently accurate to register a hemispheric pattern.

In conclusion, the coherence of KD seasonality in the Northern Hemisphere extra-tropics suggests that research efforts should focus on identifying environmental variables that connect the disease across distant regions. Analysis of aerosols and tropospheric wind patterns may be a fruitful avenue of investigation.

## Supporting Information

Table S1 (DOCX)
