# Supplementary material for: Seasonality of Kawasaki Disease: A Global Perspective
Source: PLoS One. 2013 Sep 18;8(9):e74529. doi: 10.1371/journal.pone.0074529 (PMC3776809; doi:10.1371/journal.pone.0074529)
Supplement: Table S1 — (DOCX) [file pone.0074529.s001.docx]

Supporting Document 1. International Kawasaki Disease Climate Consortium

| Contributors | Affiliations |
| --- | --- |
| N. Dahdah, M. Gibbon, R. Scuccimarri | Sainte-Justine University Hospital Center,  Montreal Children's Hospital/McGill University Health Centre, Montreal, Canada |
| B. McCrindle, C. Manlhiot | The Hospital for Sick Children, University of Toronto, Toronto, Ontario, Canada |
| C-J. Jin, L-H. Jin, Z-Y Jin, J-H Piao, Y Zhou | Department of Pediatrics, First Affiliated Hospital, Jilin University, Jilin, China |
| F. Jiao | Dept. of Pediatrics, The Shaanxi Provincial People's Hospital of Xi, Xi’an, Shaanxi, China |
| G-Y. Huang | Pediatric Heart Center, Children’s Hospital of Fudan University, Shanghai, China |
| E. Salo | Helsinki University Central Hospital, Helsinki, Finland |
| R. Cimaz, S. Di-Filippo, J-C. Lega | Department of Pediatric Cardiology, Louis Pradel Hospital, Lyons, France |
| R. Aulakh, S.Singh, D. Suri | Pediatric Allergy Immunology Unit, Advanced Pediatrics Centre, Post Graduate Institute of Medical Education and Research, Chandigarh, India |
| M.Bar-Meir | Pediatrics and Infectious diseases, Shaare-Zedek Medical Center, Jerusalem, Israel |
| R. Cimaz | Department of Pediatric Rheumatology, Anna Meyer Children’s Hospital, Florence, Italy |
| Y. Nakamura, R. Uehara | Department of Public Health Jichi Medical University, Shimotsuke-shi, Japan |
| K-Y. Lee | Department of Pediatrics, The Catholic University of Korea, Daejeon St. Mary's Hospital, Daejeon, Korea |
| J-W Han | Department of Pediatrics, The Catholic University of Korea, Uijeongbu St. Mary's Hospital, Uijeoungbu, Korea |
| M-K.Han, Y-M. Hong*, G-Y. Jang**, D-S. Kim***, H-D. Lee****, J-K. Lee and I-S. Park*****, M-S. Song******, S-W. Yun******* | Department of Pediatrics, University of Ulsan, Gangneung Asan Hospital, *Department of Pediatrics, Ewha Womans University Hospital, **Department of Pediatrics, Korea University Hospital, ***Department of Pediatrics, Yonsei University College of Medicine, Severance Children's Hospital, ****Department of Pediatrics, Pusan National University Hospital, *****University of Ulsan, Asan Medical Center,******Department of Pediatrics, Inje Univeristy, Paik Hospital, *******Department of Pediatrics, Chung-Ang Univeristy Hospital, Seoul, Korea |
| W.B. Breunis, T.W. Kuijpers, C.E. Tacke | Division of Pediatric Hematology, Immunology and Infectious diseases, Emma Children's Hospital, Academic Medical Center, Amsterdam, Netherlands |
| G. Lyskina, O. Shirniskaya, A. Torbyak | Sechenov First Moscow State Medical University, Moscow, Russia |
| L. Bregel, T.Soldatova, V.Subbotin, | Irkutsk State Academia of Continuing Medical Education, Kawasaki Disease Center, Irkutsk Regional Children"s Hospital, Irkutsk Regional Hospital, Irkutsk, Russia |
| J. Anton, F. Prada*, S. Ricart, R. Bou | Pediatric Rheumatology Unit, and *Cardiology Department, Hospital Sant Joan de Déu and  Universitat de Barcelona, Barcelona, Spain |
| S. Atalay*, E. Çiftçi, E. İnce, A. Karbuz, H.Özdemir | Department of Pediatric Infectious Diseases and *Department of Pediatric Cardiology, Ankara University Medical School, Ankara, Turkey |
| A. Harnden, M. Levin*, R. Mayon-White, R. Tulloh**, C. Michie***, V. Wright* | Department of Primary Health Care Sciences, University of Oxford, Oxford *Pediatrics Faculty of Medicine, Imperial College, London, ** Bristol Royal Hospital for Children, Bristol ***Ealing Hospital NHS Trust, London, United Kingdom |
| A.L. Baker, J.W. Newburger | Children's Hospital Boston, Boston, Massachusetts, United States of America |
| N. Innocentini, S. T. Shulman | Division of Infectious Diseases, Ann and Robert H. Lurie Children's Hospital of  Chicago, Chicago, Illinois, United States of America |
| M. Anderson, S. Dominguez, M. Glode | Pediatric Infectious Disease Children's Hospital Colorado, Denver, Colorado, United States of America |
| A. Arrieta | Pediatric Infectious Diseases, Children's Hospital of Orange County, Orange, California, United States of America |
| C. Dozal, W. Mason | Children's Hospital of Los Angeles, Los Angeles, California, United States of America |
| J. Beck | Department of Pediatrics, Loma Linda University Medical Center, Riverside, California, United States of America |
| J. C. Burns, A. Tremoulet, S. Fernandez | Department of Pediatrics, UCSD School of Medicine, San Diego, California, United States of America |
| C.M. Magalhaes, R. Pratesi | Dept. of Pediatrics, Brasilia University School of Medicine, Brasilia, Brazil |
| Y-F. Cheung | Department of Pediatrics and Adolescent Medicine, Queen Mary Hospital, Hong Kong, China |
| M. Reyes | Departamento de Microbiología, Pontificia Universidad Javeriana, Bogota, Colombia |
| N. Advani | University of Indonesia, Jakarta Pusat, Jakarta, Indonesia |
| O. Olugbuyi, R. Pierre | Department of Child and Adolescent Health, University of the West Indies, Kingston, Jamaica |
| E. Castaño, D. Estripeaut and X. Sáez-Llorens | University of Panama School of Medicine, Hospital del Niño, Panama City, Panama |
| C. K. Chen, T. L. J. Choo, T. H. Tan, K. Y. Wong | Cardiology Service, Department of Pediatric Subspecialties KK Women's & Children's Hospital, Singapore |
| H-C. Kuo, M-T. Lin*, M-H. Wu* | Department of Pediatrics, Kaohsiung Chang Gung Memorial Hospital, *Department of Pediatrics, National Taiwan University Hospital, Taipei & Kaohsiung, Taiwan |
| R. Sittiwangkul | Division of Cardiology, Department of Pediatrics, Chiang Mai University, Chiangmai, Thailand |
| M. Melish | Department of Pediatrics, Kapiolani Medical Center, Honolulu, Hawaii, United States of America |
| D. Burgner, M. Odam | Murdoch Children’s Research Institute and Department of Pediatrics, University of Melbourne, Melbourne, Perth Australia |
| A. Salgado, G. Soza | Dr. Hernan Henriquez Aravena Hospital, Temuco, Araucania, Chile |
| J. Doran, P. Heaton*, N. Wilson** | Taranaki Base Hospital, *Paediatric Department Yeovil District Hospital, **Department of Pediatric Cardiology, Starship Hospital, Auckland, New Zealand |
| B. Eley, D. Moore* | Pediatric Infectious Diseases Unit, Red Cross War Memorial Children's Hospital and Department of Pediatrics and Child Health, University of Cape Town, *Respiratory and Meningeal Pathogens Research Unit, University of the Witwatersrand, Cape Town & Johannesburg, South Africa |
